# The Presence of Biomarker Enzymes of Selected Scleractinian Corals of Palk Bay, Southeast Coast of India

**DOI:** 10.1155/2014/684874

**Published:** 2014-08-18

**Authors:** R. Anithajothi, K. Duraikannu, G. Umagowsalya, C. M. Ramakritinan

**Affiliations:** Department of Marine and Coastal Studies, Madurai Kamaraj University, Tamilnadu 623526, India

## Abstract

The health and existence of coral reefs are in danger by an increasing range of environmental and anthropogenic impacts. The causes of coral reef decline include worldwide climate change, shoreline development, habitat destruction, pollution, sedimentation and overexploitation. These disasters have contributed to an estimated loss of 27% of the reefs. If the current pressure continues unabated, the estimated loss of coral reef will be about 60% by the year 2030. Therefore, the present study was aimed to analyze the enzymes involved in stress induced by coral pathogen and its resistance. We focused on the enzymes involved in melanin synthesis pathway (phenoloxidase (PO) and peroxidases (POD)) and free radical scavenging enzymes (super oxide dismutase (SOD), catalase (CAT)) and glutathione peroxidase (Gpx) in selected scleractinian corals such as *Acropora formosa, Echinopora lamellosa, Favia favus, Favites halicora, Porites* sp., and *Anacropora forbesi.* Overall, PO activity of coral was significantly lower than that of zooxanthellae except for *Favia favus.* Coral colonies with lower PO and POD activities are prone to disease. Maximum antioxidant defensive enzymes were observed in *Favia favus* followed by *Echinopora lamellose.* It is concluded that assay of these enzymes can be used as biomarkers for identifying the susceptibility of corals towards coral bleaching induced by pathogen.

## 1. Introduction

The coral holobiont is comprised not only of coral animal and endosymbiotic dinoflagellates (zooxanthellae) but also of microbial communities such as bacteria, archaea, and fungi as well as numerous viruses [1, 2]. As a primitive organism, corals do have an innate immune system similar to that of other invertebrates [3–6] but do not possess the adaptive immune system of vertebrates. The defensive mechanism of the immune system plays a vital role in prevention of infection and in the maintenance of tissue integrity of corals [7, 8]. The recognition of the coral defensive system involved in immune responses like other invertebrates includes Toll-like receptors [9] and the synthesis of melanin. The melanin synthesis is activated by phenoloxidase, immune cells and antioxidants, and peroxidase [10–13]. During PO pathways, the cytotoxic intermediates lead to inflammatory defense [5, 14].

Prophenoloxidase is an important enzyme and a key component of innate immunity system of corals [15]. Pathogenic microbes trigger the synthesis of melanin through the cleavage of prophenoloxidase (PPO) into phenoloxidase (PO). Various phenoloxidases catalyse monophenol hydroxylation and diphenol oxidation as well as autocatalytic reactions which frequently lead to the formation of the pigment, melanin [14–16]. Oxidative burst is resulting in upregulation of PPO activity because PPO activity is the primary sources of oxidative stress during an invertebrate immune response [17, 18]. This leads to the production of antioxidant enzymes such as SOD, CAT, POD, and GPx. Our study focused on the biomarker enzymes involved in defensive mechanism of selected corals of Palk Bay situated at southeast of India.

## 2. Materials and Methods

### 2.1. Tissue Homogenate for Enzyme Assays

Six different coral specimens were collected from three different locations, that is, from villundi theertham (lat.: 9°17′33.81′′N, long.: 79°12′ 46.69′′E), Pamban (lat.: 9°17′1.21′′N, long.: 79°12′44.20′′E), and Olaikuda (lat.: 9°18′30.12′′N, long.: 79°20′ 4.44′′E) of the Palk Bay, southeast coast of India. Specimens were collected during low tide in the early morning in the month of April 2012. These were identified based on the morphological features and keys observed during collection of samples. The identified corals were (1)* Acropora formosa *(AC), (2)* Echinopora lamellosa* (EL), (3)* Favia favus *(FF), (4)* Favites halicora *(FH), (5)* Porites *sp. (POR), and (6)* Anacropora forbesi* (AN). Coral fragments of about 2 cm^2^ were collected using a hammer, stored in an ice pack, and immediately transported to the laboratory.

The coral samples were crushed using sterile sea water and the homogenate was obtained by centrifugation at 15,000 RPM for 15 minutes to separate the supernatant and pellets. The supernatant thus obtained was considered as substrate for the analysis of protein and enzyme activities of coral host [19] and the pellet was considered to be a substrate for analysis of protein and enzyme activities of zooxanthellae. The pellets containing the zooxanthellae were suspended in 2 mL of 100 mmol^−1^ phosphate buffer (pH-7.0) and were dissolved by sonication for 30 minutes in an ice bath. Triton X-100 (0.05%) solution was added to the sonicated suspension and kept for 10 minutes at room temperature and then the suspension was centrifuged at 14,000 rpm for 30 minutes and used as the zooxanthellae extract for protein and enzyme assays. The protein was estimated using standardized protocols prescribed by Lowry et al., 1951 [20].

### 2.2. Peroxidase and Phenoloxidase

Peroxidase activity was assayed spectrophotometrically (SPECRTA max M2e) at 470 nm using guaiacol as a phenolic substrate with hydrogen peroxidase [21]. Five milliliters of the assay mixture for the peroxidase activity comprised 35 *μ*L of phosphate buffer (10 mmol^−1^ pH 6.0), 10 *μ*L of sample extract (coral tissue and zooxanthellae solution of six different coral samples), 40 *μ*L of guaiacol (25 mmol^−1^), and 25 *μ*L of H_2_O_2_ (20 mmol^−1^). The reaction was stopped by adding 5% H_2_SO_4_^∙^ POD activity was expressed as U*·*mg^−1^ protein. Units were calculated using a molar absorptivity of 2.66 × 104 M^−1 ^cm^−1^ for tetraguaiacol or 3,3′-dimethoxy-4,4′-biphenoquinone [22]. Phenoloxidase activity was calculated using the same procedure followed for the analysis of peroxidase but without peroxide.

### 2.3. Superoxide Dismutase Activity

Quantification of SOD activity was based on the ability of SOD to inhibit the reduction of NBT by superoxide [23]. Two hundred microlitres of enzyme extract was added to a tube containing 0.2 mL of 0.1 M EDTA solution, 0.3 mM sodium cyanide, and 0.1 mL of 1.5 mM NBT. Then, 0.05 mL of 0.12 mM riboflavin was added at zero timed intervals and then all the tubes were incubated in a light box for 12 minutes and absorbance was recorded at 560 nm using SPECRTA max M2e. One unit of SOD was defined as the enzyme causing half the maximum inhibition of NBT reduction and SOD activity was expressed as U*·*mg^−1^ protein.

### 2.4. Catalase Activity

Catalase activity was estimated by the method described by Aebi, 1984 [24]. Catalase activity was observed by measuring the decrease in H_2_O_2_ concentration at 240 nm. Working solution of 340 *μ*L of 100 mM phosphate buffer with pH 7.0, 10 mM H_2_O_2_, and 660 *μ*L of sample extract were mixed in a cuvette. The change in absorbance per minute at 240 nm was calculated. Enzyme activity was expressed in units of CAT activity and was expressed as U*·*mg^−1^ protein. One unit of CAT activity was defined as the amount of enzyme needed to reduce 1 *μ*M H_2_O_2_ *·*min^−1^ [24].

### 2.5. Glutathione Peroxidase Activity

Glutathione peroxidase (GPx) activity was determined using a slightly modified protocol of Rotruck et al., 1973 [25]. The reaction mixture was prepared by adding 500 *μ*L of tissue homogenate, 200 *μ*L of phosphate buffer (0.4 M pH-7), 200 *μ*L of 0.45 mM EDTA, 100 *μ*L of 10 mM sodium azide, and 200 *μ*L of GSH solution followed by 0.1 mL H_2_O_2_. The contents were incubated for 10 min at 37°C. In this mixture, 0.4 mL of 10% TCA was added to stop the reaction and the mixture was centrifuged at 3200 ×g for 20 min. The pellet was discarded and 0.5 mL of DTNB was added to the supernatant. The supernatant was assayed spectrophotometrically (SPECRTA max M2e) at 340 nm. One unit of glutathione peroxidase activity was defined as the amount of enzyme that oxidizes 1 *μ*mol of reduced glutathione and GPx activity was expressed as U*·*mg^−1^ protein.

### 2.6. Statistical Analysis

Data were analyzed by one-way analysis of variance (ANOVA) with the Graph-Pad Prism 5 software and the least significant differences were compared at *P* < 0.05.

## 3. Results

### 3.1. Phenoloxidase Activity

**Figure 1:**
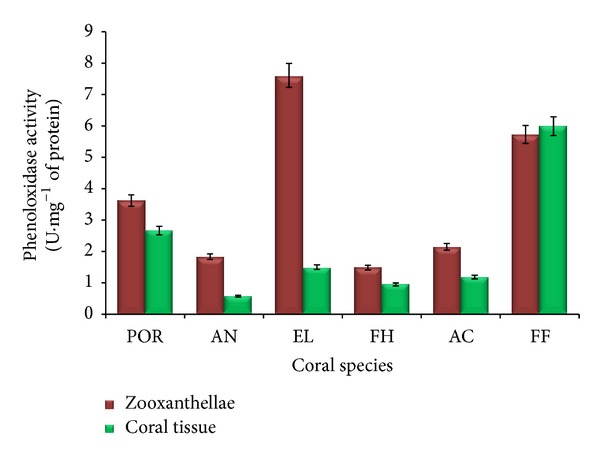
Phenoloxidase activity of coral tissue and zooxanthellae (*n* = 3). POR:* Porites *sp., AN:* A. forbesi*, EL:* E. lamellosa,* FH:* F. halicora*, AC:* A. formosa*, and FF:* F. favus.*

Phenoloxidase activity of both zooxanthellae and coral tissues of six healthy coral samples collected from the Palk Bay was presented in Figure 1. The phenoloxidase activity of zooxanthellae of* Porites* sp.,* A. forbesi*,* E. lamellosa*,* F. halicora*,* A. formosa,* and* F. favus *was 3.6, 1.8, 7.6, 1.5, 2.0, and 5.7 U*·*mg^−1^protein, respectively. Maximum PO activity was noticed in zooxanthellae of* E. lamellosa* and the minimum was noticed in* F. halicora* zooxanthellae extract. Maximum PO activities of zooxanthellae are exhibited by* E. lamellosa*.

The phenoloxidase activity of coral tissues of six different corals such as* Porites* sp.,* A. forbesi*,* E. lamellosa*,* F. halicora*,* A. formosa,* and* F. favus *was 2.7, 0.5, 1.5, 0.95, 1.2, and 5.9 U*·*mg^−1^ protein, respectively. Minimum and maximum PO activities were reported in the coral tissues of* A. forbesi* and* F. favus*, respectively (Figure 1). Maximum PO activity of coral tissue was exhibited by* F. favus*. Except in* F. favus*, the PO activity of zooxanthellae was significantly higher than coral tissues in all species. The PO activity of zooxanthellae was significantly higher than that of their respective coral tissues except* F. favus*.

### 3.2. Peroxidase Activity

**Figure 2:**
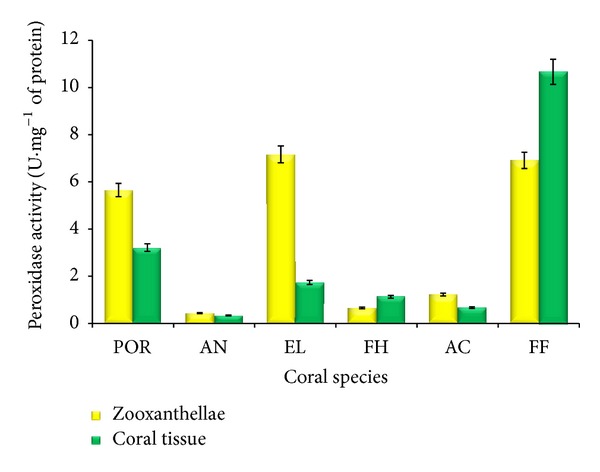
Peroxidase activity of coral tissue and zooxanthellae (*n* = 3). POR:* Porites *sp., AN:* A. forbesi*, EL:* E. lamellosa,* FH:* F. halicora*, AC:* A. formosa*, and FF:* F. favus.*

Peroxidase (POD) activity of both zooxanthellae and coral tissues of six different coral species is presented in Figure 2. The peroxidase activity of zooxanthellae of* Porites* sp.,* A. forbesi*,* E. lamellosa*,* F. halicora*,* A. formosa,* and* F. favus *was 5.65, 0.43, 7.1, 0.65, 1.22, and 6.91 U*·*mg^−1^ protein, respectively. Maximum POD activity was observed in zooxanthellae of* E. lamellosa* and the minimum was observed in zooxanthellae of* A. forbesi* (Figure 2). POD activity of* A. forbesi* was 17-fold higher than that of* E. lamellosa*.

The peroxidase activity of coral tissues of six different coral species such as* Porites* sp.,* A. forbesi*,* E. lamellosa*,* F. halicora*,* A. formosa,* and* F. favus *was 3.21, 0.34, 1.73, 1.13, 0.67, and 10.7 U*·*mg^−1^ protein, respectively. Maximum POD activity of coral tissues was found in* F. favus* and the minimum activity was found in* A. forbesi *(Figure 2). The POD activity of* F. favus* was 32-fold higher than that of* A. forbesi* (Figure 2).

Significant increase in peroxidase activity was observed in zooxanthellae extracts when compared with coral tissue extracts of all coral samples except* F. favus *(Figure 2).

### 3.3. Antioxidant Defensive Factors of Corals

The three major antioxidant enzymes are the superoxide dismutase (SOD) which produces H_2_O_2_ by converting O_2_^•−^, then, the enzyme catalase (CAT) that splits H_2_O_2_ to oxygen and water, and the third antioxidant enzyme, that is, glutathione peroxides (GPx), that converts H_2_O_2_ and organic peroxides. Increased level of ROS activates these three antioxidant enzymes.

#### 3.3.1. Superoxide Dismutase Activity

**Figure 3:**
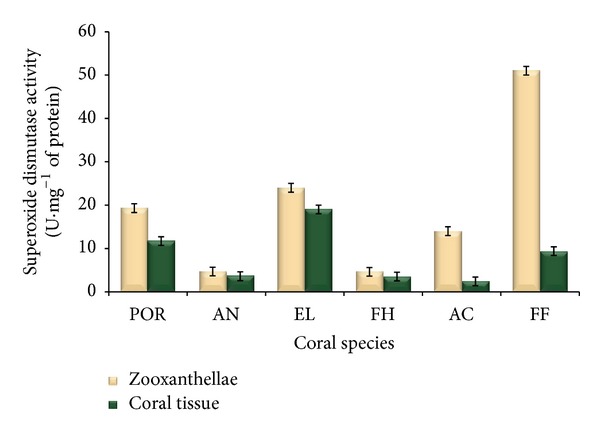
SOD activity of coral tissue and zooxanthellae of 6 coral species (*n* = 3). POR:* Porites *sp., AN:* A. forbesi*, EL:* E. lamellosa*, FH:* F. halicora*, AC:* A. formosa*, and FF:* F. favus.*

Superoxide dismutase activity of both zooxanthellae and coral tissues of six different coral species is presented in Figure 3. SOD activity of zooxanthellae extract of* Porites* sp.,* A. forbesi*,* E. lamellosa*,* F. halicora*,* A. formosa,* and* F. favus *was 19.4, 4.7, 24, 4.7, 14.1, and 51.4 U*·*mg^−1^ protein, respectively. Maximum SOD activity was noticed in the zooxanthellae extract of* F. favus*, while* A. forbesi* and* F. halicora* exhibited low SOD activity (Figure 3).

SOD activity of coral tissues of* Porites* sp.,* A. forbesi*,* E. lamellosa*,* F. halicora*,* A. formosa,* and* F. favus *was 11.7, 3.3, 19.3, 3.5, 2.4, and 9.4 U*·*mg^−1^ protein, respectively. The coral tissues of* A. formosa* exhibited minimum SOD activity and* E. lamellosa* tissues exhibited maximum activity (Figure 3). SOD activity of* E. lamellosa* was 8-fold higher than that of* A. formosa* and 5-fold higher than that of* Porites* sp.

SOD activity of* F. favus* was found to be 11-fold higher than that of* A. forbesi* and* F. halicora* followed by 5- and 4-fold higher activity than* E. lamellosa* and* Porites *sp*.,* respectively. Except* A. forbesi* and* F. halicora*, the SOD activity significantly varied between zooxanthellae and coral tissue in all species.

#### 3.3.2. Catalase Activity

**Figure 4:**
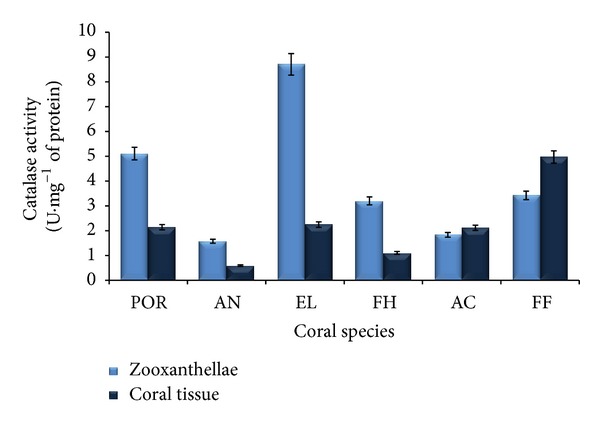
Catalase activity of coral tissue and zooxanthellae (*n* = 3). POR:* Porites *sp., AN:* A. forbesi*, EL:* E. lamellosa*, FH:* F. halicora*, AC:* A. formosa*, and FF:* F. favus.*

Catalase (CAT) activities of zooxanthellae and coral tissues of six different coral species have been presented in Figure 4. CAT activity of zooxanthellae of* Porites *sp.*, A. forbesi, E. lamellosa, F. halicora, A. formosa,* and* F. favus* was 5.1, 1.6, 8.7, 3.2, 1.8, and 3.4 U*·*mg^−1^ protein, respectively (Figure 4). CAT activity of zooxanthellae extract of* E. lamellosa* exhibited maximum activity while zooxanthellae of* A. forbesi* exhibited minimum catalase activity (Figure 4). CAT activity of* E. lamellosa* was 5.4-fold higher than that of* A. forbesi* and 3.2-fold higher than that of* Porites *sp.

Similarly, CAT activity of coral tissues of* Porites* sp.,* A. forbesi*, *E. lamellosa*,* F. halicora*,* A. formosa,* and* F. favus *was 2.1, 0.6, 2.2, 1.1, 2.1, and 5.0 U*·*mg^−1^ protein, respectively (Figure 4). The coral tissues of* A. forbesi* and* F. favus *exhibited minimum and maximum CAT activities, respectively (Figure 4). CAT activity of tissues of* A. forbesi* was 8.3-fold higher than that of* F. favus *and 3.7-fold higher than that of* E. lamellosa*.

Comparing CAT activity between coral zooxanthellae and coral tissues of the six different species, it was observed that, except* A. formosa* and* F. favus*, CAT activity was higher in zooxanthellae than that of coral tissues in all species.

#### 3.3.3. Glutathione Peroxidase

**Figure 5:**
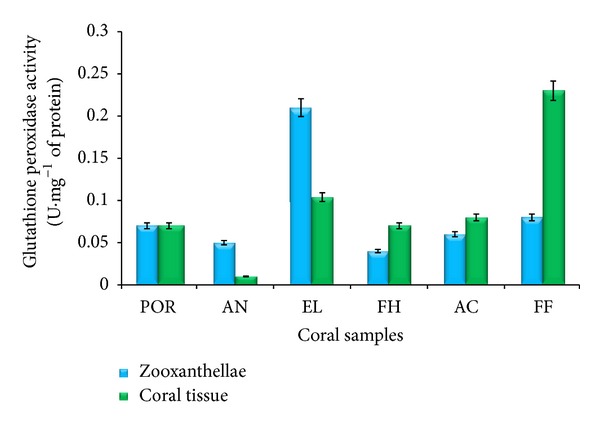
Glutathione peroxidase activity of coral tissue and zooxanthellae (*n* = 3). POR:* Porites *sp., AN:* A. forbesi*, EL:* E. lamellosa*, FH:* F. halicora*, AC:* A. formosa*, and FF:* F. favus.*

Like SOD and CAT activities, glutathione peroxidase (GPx) activities of both zooxanthellae and coral tissues of six different corals were also estimated and presented in Figure 5. GPx activity of zooxanthellae of* Porites* sp.,* A. forbesi*,* E. lamellosa*,* F. halicora*,* A. formosa,* and* F. favus *was 0.07, 0.05, 0.21, 0.04, 0.06, and 0.08 U*·*mg^−1^ protein, respectively.

The zooxanthellae of* E. lamellosa* and* F. halicora* exhibited maximum and minimum GPx activities, respectively (Figure 5). Comparatively, GPx activity of* E. lamellose* was 5.3-fold higher than that of* F. halicora* zooxanthellae. There was no significant difference between GPx activity of zooxanthellae of* Porites sp.* and* F. favus* while the remaining four species showed significant variations in activity.

Similar to GPx activity of zooxanthellae, the GPx activity of coral tissues has been presented in Figure 5 and the activity was 0.07, 0.01, 0.1, 0.07, 0.08, and 0.23 U*·*mg^−1^ protein, respectively. The tissues of* A. forbesi* exhibited minimum GPx activity and* F. favus* tissues exhibited maximum activity (Figure 5).

The GPx activities of both zooxanthellae and the coral tissues of* Porites *sp. were similar. But the GPx activity of coral tissues of* A. forbesi* and* E. lamellosa* was less than that of respective coral zooxanthellae activity. However, the activity was more in coral tissues of* F. halicora, A. formosa,* and* F. favus* than the respective coral zooxanthellae.

## 4. Discussion

As phenoloxidase plays a vital role in defensive mechanism of invertebrates, the presence of PO activity in all 6 coral species indicates the presence of baseline level of antimicrobial defense [13]. Generally, invertebrate animals with decreased phenoloxidase (PO) activity are more susceptible to disease [26–28]. In the present study,* A. forbesi, F. halicora, *and* A. formosa* showed lowest phenoloxidase activity. It reveals that these coralsare susceptible to bleaching, thermal stress, and diseases. At the same time,* F. favus, E. lamellose,* and* Porites *sp. indicated higher disease resistance towards pathogens [13]. The low level of PO activity in* A. formosa *and* A. forbesi* showed that branching corals are more susceptible to diseases. Similar results were observed in the study of susceptible ranking of 15 scleractinian corals by Palmer et al. [13].

Peroxidase activity induction includes oxidation of substrates and cytotoxic molecules [14, 17] which act as antimicrobial agents [29]. In most cases, peroxidase provides resistance to the corals against fungal pathogens as in case of* Aspergillosis* [30]. In our findings, the maximum peroxidase activity was observed in the tissue of* Favia favus* and zooxanthellae of* E. lamellosa* and the lower activity was observed in* A. forbesi*. Pathogen recognition results in apoplastic generation of superoxide (O^−1^), hydrogen peroxide (H_2_O_2_), and hydroxyl radicals [31], thereby initiating the upregulation of antioxidant enzymes. ROS production is provided with an array of antioxidant enzymes, which either convert O_2_^•−^ to H_2_O_2_ (SOD), convert H_2_O_2_ to water and oxygen (CAT), or use H_2_O_2_ to oxidize substrates (glutathione peroxidase) [31]. The principal findings reveal that corals such as* F. favus* and* E. lamellosa* have strong resistance towards bleaching and disease and, at the same time,* Anacropora forbesi* are more susceptible to bleaching and diseases [13]. The SOD activities of zooxanthellae were too high when compared with the SOD activities of coral tissue of all coral samples analyzed in this study. The findings suggested the production of SOD by coral host in response to O_2_ production during photosynthesis of its particular host.

The maximum catalase activity was observed in zooxanthellae compared to coral tissue. This may be due to the conversion of superoxide ion produced during photosynthesis into hydroxyl ion which was denoted by the increase in GPx and SOD activity, which in turn resulted in hydrogen peroxide free radical in the zooxanthellae. Hence, the increase in CAT activities was observed as a result of increased H_2_O_2_ concentrations in the zooxanthellae [32].

Glutathione peroxidase (GPx) plays a vital role in cleaving H_2_O_2_ as a compensatory mechanism under severe oxidative challenge where the catalase activity may be inhibited [33, 34]. The antioxidant enzymes such as SOD, CAT, and GPx are reported to be increased during thermal and salinity stress in response to ROS generated during these stresses [35].

## 5. Conclusion

The present study focused on the enzymes involved in the coral resistance among the selected coral species of the Palk Bay region, southeast coast of India. The results of this study described that the corals* E. lamellosa*, followed by* F. favus* and* Porites *sp., were exhibited maximum activities of defensive enzymes. Accordingly, it was concluded that these corals were highly resistant towards the coral pathogens. While the corals of* A. formosa*,* F. halicora,* and* A. forbesi* exhibited minimum activities of the defensive enzymes indicating susceptibility to coral pathogens, these susceptible corals are prone to disease and should be prioritized to prevent transmittance of disease.

## References

[1] AnithaJothi R, Umagowsalya G, Duraikannu K, Ramkumar B, Santhana krishnan M, Ramakrtinan CM (2013). Isolation and characterization of microbes associated with White pox diseased coral (Acroporidae) from Palk Bay, southeast coast of India. *Asian Journal of Marine Science*.

[2] Anithajothi R, Nagarani N, Umagowsalya G, Duraikannu K, Ramakritinan CM (2014). Screening, isolation and characterization of protease producing moderately halophilic microorganism *Halomonas meridiana* associated with coral mucus. *Toxicological & Environmental Chemistry*.

[3] Dunn SR (2009). Immunorecognition and immunoreceptors in the Cnidaria. *Invertebrate Survival Journal*.

[4] Mydlarz LD, Jacobs RS (2006). An inducible release of reactive oxygen radicals in four species of gorgonian corals. *Marine and Freshwater Behaviour and Physiology*.

[5] Mydlarz LD, McGinty ES, Harvell CD (2010). What are the physiological and immunological responses of coral to climate warming and disease?. *Journal of Experimental Biology*.

[6] Reed KC, Muller EM, van Woesik R (2010). Coral immunology and resistance to disease. *Diseases of Aquatic Organisms*.

[7] Cooper EL (2003). Comparative immunology. *Current Pharmaceutical Design*.

[8] Stedman TL (2000). *Stedman’s Medical Dictionary*.

[9] Miller DJ, Hemmrich G, Ball EE (2007). The innate immune repertoire in Cnidaria—ancestral complexity and stochastic gene loss. *Genome Biology*.

[10] Cooper E (2008). From Darwin and Metchnikoff to Burnet and beyond. *Contributions to Microbiology*.

[11] Mydlarz LD, Holthouse SF, Peters EC, Harvell CD (2008). Cellular responses in sea fan corals: granular amoebocytes react to pathogen and climate stressors. *PLoS ONE*.

[12] Palmer CV, Mydlarz LD, Willis BL (2008). Evidence of an inflammatory-like response in non-normally pigmented tissues of two scleractinian corals. *Proceedings of the Royal Society B: Biological Sciences*.

[13] Palmer CV, Bythell JC, Willis BL (2010). Levels of immunity parameters underpin bleaching and disease susceptibility of reef corals. *The FASEB Journal*.

[14] Nappi AJ, Christensen BM (2005). Melanogenesis and associated cytotoxic reactions: applications to insect innate immunity. *Insect Biochemistry and Molecular Biology*.

[15] Cerenius L, Lee BL, Söderhäll K (2008). The proPO-system: pros and cons for its role in invertebrate immunity. *Trends in Immunology*.

[16] Cerenius L, Kawabata S, Lee BL, Nonaka M, Söderhäll K (2010). Proteolytic cascades and their involvement in invertebrate immunity. *Trends in Biochemical Sciences*.

[17] Nappi AJ, Ottaviani E (2000). Cytotoxicity and cytotoxic molecules in invertebrates. *BioEssays*.

[18] Sadd BM, Siva-Jothy MT (2006). Self-harm caused by an insect's innate immunity. *Proceedings of the Royal Society B: Biological Sciences*.

[19] Higuchi T, Fujimura H, Arakaki T Oomori activities of antioxidant enzymes (SOD and CAT) in the coral *Galaxea fascicularis* against increased hydrogen peroxide concentrations in seawater.

[20] Lowry OH, Rosebrough NJ, Farr AL, Randall RJ (1951). Protein measurement with the Folin phenol reagent. *The Journal of Biological Chemistry*.

[21] Díaz J, Bernal A, Pomar F, Merino F (2001). Induction of shikimate dehydrogenase and peroxidase in pepper (*Capsicum annuum* L.) seedlings in response to copper stress and its relation to lignification. *Plant Science*.

[22] Maehly AC (1954). *Methods of Biochemical Analysis*.

[23] Beauchamp C, Fridovich I (1971). Superoxide dismutase: Improved assays and an assay applicable to acrylamide gels. *Analytical Biochemistry*.

[24] Aebi H (1984). Catalase *in vitro*. *Methods in Enzymology*.

[25] Rotruck JT, Pope AL, Ganther HE, Swanson AB, Hafeman DG, Hoekstra WG (1973). Selenium: biochemical role as a component of glatathione peroxidase. *Science*.

[26] Butt D, Raftos D (2008). Phenoloxidase-associated cellular defence in the Sydney rock oyster, Saccostrea glomerata, provides resistance against QX disease infections. *Developmental and Comparative Immunology*.

[27] Mucklow PT, Vizoso DB, Jensen KH, Refardt D, Ebert D (2004). Variation in phenoloxidase activity and its relation to parasite resistance within and between populations of *Daphnia magna*. *Proceedings of the Royal Society B: Biological Sciences*.

[28] Newton K, Peters R, Raftos D (2004). Phenoloxidase and QX disease resistance in Sydney rock oysters (*Saccostrea glomerata*). *Developmental and Comparative Immunology*.

[29] Caruso C, Chilosi G, Leonardi L (2001). A basic peroxidase from wheat kernel with antifungal activity. *Phytochemistry*.

[30] Mydlarz LD, Harvell CD (2007). Peroxidase activity and inducibility in the sea fan coral exposed to a fungal pathogen. *Comparative Biochemistry and Physiology*.

[31] Torres MA, Jones JDG, Dangl JL (2006). Reactive oxygen species signaling in response to pathogens. *Plant Physiology*.

[32] Higuchi T, Fujimura H, Arakaki T, Oomari T Activities of antioxidant enzymes (SOD and CAT) in the coral *Galaxea fasicularis* against increased peroxide concentrations in seawater.

[33] Regoli F, Benedetti ME, Giuliani ME, Amiard-Triquet C, Rainbow C, Romeo PS (2011). Antioxidant defenses and acquisition of tolerance to chemical stress. *Tolerance to Environmental Contaminants*.

[34] Regoli F, Giuliani ME, Benedetti M, Arukwe A (2011). Molecular and biochemical biomarkers in environmental monitoring: a comparison of biotransformation and antioxidant defense systems in multiple tissues. *Aquatic Toxicology*.

[35] de Almeida EA, Di Mascio P (2011). Hypometabolism and antioxidative defense systems in marine invertebrates hypometabolism. *Strategies of Survival in Vertebrates and Invertebrates*.

